# A Novel 6-Benzyl Ether Benzoxaborole Is Active against Mycobacterium tuberculosis *In Vitro*

**DOI:** 10.1128/AAC.01205-17

**Published:** 2017-08-24

**Authors:** Nipul Patel, Theresa O'Malley, Yong-Kang Zhang, Yi Xia, Bjorn Sunde, Lindsay Flint, Aaron Korkegian, Thomas R. Ioerger, Jim Sacchettini, M. R. K. Alley, Tanya Parish

**Affiliations:** a TB Discovery Research, Infectious Disease Research Institute, Seattle, Washington, USA; b Anacor Pharmaceuticals, Palo Alto, California, USA; c Texas A&M University, College Station, Texas, USA

**Keywords:** antimicrobial, antitubercular, drug resistance

## Abstract

We identified a novel 6-benzyl ether benzoxaborole with potent activity against Mycobacterium tuberculosis. The compound had an MIC of 2 μM in liquid medium. The compound was also able to prevent growth on solid medium at 0.8 μM and was active against intracellular bacteria (50% inhibitory concentration [IC_50_] = 3.6 μM) without cytotoxicity against eukaryotic cells (IC_50_ > 100 μM). We isolated resistant mutants (MIC ≥ 100 μM), which had mutations in Rv1683, Rv3068c, and Rv0047c.

## TEXT

Tuberculosis (TB) remains a serious global health problem, with an increase in the reported incidence of new infections combined with increasing levels of drug resistance (1). We are interested in finding new molecules with antitubercular activity and also in determining the mode of resistance to new agents and/or their molecular targets.

**FIG 1:**
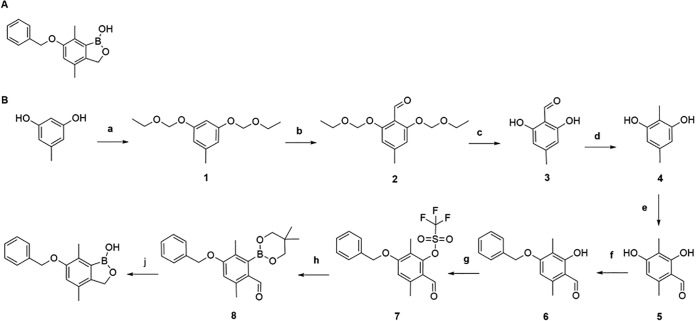
(A) Structure of 6-benzyl ether. (B) Synthetic pathway for compound. ^a^, chloromethyl ethyl ether, DIPEA, DCM, room temperature (rt), overnight; ^b^, *n*-butyl lithium, DMF, THF, 18°C, 1.5 h; ^c^, HCl, THF, rt, overnight; ^d^, sodium cyanoborohydride, THF, rt, 3 h; ^e^, phosphorus oxychloride, DMF, rt, overnight; ^f^, benzyl bromide, NaHCO_3_, KI, AcCN, 80°C, overnight; ^g^, triflic anhydride, triethylamine, DCM, rt, 3 h; ^h^, 5,5,5′,5′-tetramethyl-2,2′-bi(1,3,2-dioxaborinane), PdCl_2_(dppf)_2_, potassium acetate, 1,4-dioxane, 90°C, overnight; ^j^, sodium borohydride, THF, rt, 3 h and then HCl, water, overnight.

In screening the Anacor boron library, we identified a member of the 6-benzyl ether benzoxaborole class, 6-(benzyloxy)-4,7-dimethylbenzo[c][1,2]oxaborol-1(3H)-ol (Fig. 1; see also the supplemental material), with good *in vitro* activity against Mycobacterium tuberculosis under aerobic conditions. Briefly, we tested the compound in dimethyl sulfoxide (DMSO) as 2-fold serial dilutions against M. tuberculosis H37Rv (ATCC 25618) for 5 days in Middlebrook 7H9 medium supplemented with 10% OADC (oleic acid-albumin-dextrose-catalase) and 0.05% (wt/vol) Tween 80. Growth was monitored by optical density at 590 nm (OD_590_); the MIC was determined by fitting the growth inhibition curve using the Levenberg-Marquardt algorithm. MIC was defined as the concentration required to inhibit growth by 90% (2). The compound had an MIC of 2.0 ± 0.24 μM (*n* = 6).

The cytotoxicity of the compound was determined in HepG2 cells that were cultured in Dulbecco modified Eagle medium (DMEM), 10% fetal bovine serum (FBS), and 1× penicillin-streptomycin solution (100 U/ml). Cells were exposed to compounds for 2 days at 37°C and 5% CO_2_ (final DMSO concentration of 1%). Cell viability was measured using the CellTiter-Glo reagent (Promega) and by determining relative luminescent units (RLU). Inhibition curves were fitted using the Levenberg-Marquardt algorithm and were used to calculate the 50% inhibitory concentration (IC_50_), i.e., the concentration required to reduce cell viability by 50%. We tested the compound using either glucose or galactose as the carbon source, and the IC_50_ was >100 μM (*n* = 2) under both conditions. Therefore, we tested the compound for activity against intracellular bacteria using a luminescent strain of M. tuberculosis (3). THP-1 cells were infected overnight with M. tuberculosis at a multiplicity of infection (MOI) of 1 in complete RPMI (RPMI 1640, 10% FBS, 2 mM Corning glutagro, and 1 mM sodium pyruvate). Extracellular bacteria were removed by washing, and infected cells were seeded at 4 × 10^4^ cells per well in 96-well plates containing compounds. Compounds were tested as a 10-point, 3-fold dilution series (0.5% DMSO). Infected cells were incubated for 3 days in a humidified atmosphere of 37°C and 5% CO_2_. RLU were used as a measure of bacterial viability. Growth inhibition curves were fitted using the Levenberg-Marquardt algorithm; the IC_50_ and IC_90_ were defined as the compound concentrations that produced 50% and 90% inhibition of intracellular growth, respectively. The IC_50_ and IC_90_ were 3.6 ± 0.07 μM and 22 ± 12 μM, respectively (*n* = 2).

We tested the ability of the compound to prevent growth on solid medium. We plated aerobically cultured M. tuberculosis onto Middlebrook 7H10 plus 10% OADC containing compounds (4). Plates were incubated for 3 to 4 weeks at 37°C and growth recorded. The MIC_99_ under these conditions was 5 μM; we plated M. tuberculosis H37Rv onto solid medium containing 5× or 10× the MIC and isolated colonies after 3 to 6 weeks. Clones were tested for resistance in liquid and solid media. Four isolates with high-level resistance were confirmed with MICs of ≥100 μM. DNA isolated from these mutants was subjected to whole-genome sequencing (5). Several single nucleotide polymorphisms were identified (Table 1) and confirmed by PCR amplification and sequencing.

**TABLE 1:** Profile of resistant mutants^*d*^

Mutant isolate^*a*^	MIC_99_ (μM)^*b*^	Rv0047c^*c*^	Rv3068c^*c*^	Rv1683^*c*^
RM1	100	wt	wt	L341P
RM2	>100	E128*	T351A	wt
RM3	>100	wt	wt	M200I A201T
RM4	100	E128*	T351A	wt

a Resistant mutants were isolated on solid medium.

b MIC_99_ was calculated on solid medium (4).

c The SNPs listed in the table were identified by whole-genome sequencing and confirmatory PCR/sequence in each strain.

d wt, wild type. * is a stop codon.

Two of the four strains had mutations in *Rv1683*, while the other two had mutations in *Rv0047c* and *Rv3068c*. The mutations in *Rv0047c* would result in a premature stop codon, while the mutations in *Rv3068c* would result in a threonine to alanine change. The *Rv0047c* gene is located upstream of *ino1*, which is involved in phosphatidylinositol metabolism and is required for growth on inositol (6). *Rv0047c* is proposed to be cotranscribed with *ino1*, suggesting a link with inositol metabolism. Therefore, we determined if addition of inositol had any effect on the compound activity, but we saw no shift in MIC (range, 5.4 to 5.9 μM with 6.25 to 100 μM inositol). We also tested l-histidine supplementation but saw no difference (range, 3.2 to 3.8 μM with 10 to 100 μM inositol). Since the mutation in *Rv0047c* was linked to a mutation in *Rv3068c* in both strains with the same nonsynonymous substitution, it is possible that the two strains are siblings. The *Rv3068c* gene encodes a nonessential enzyme, PgmA, a putative phosphoglucomutase involved in glucose metabolism.

*Rv1638* encodes a possible bifunctional protein involved in catabolism and anabolism of triglycerides (TGs) (7). In Mycobacterium bovis, BCG1721 (homolog of Rv1683) is responsible for accumulation and breakdown of triglycerides stored as lipid droplets (LDs) (7). Several studies have shown TGs to be a carbon source utilized by M. tuberculosis in the nonreplicating persistence phase (8), and the buildup of TGs has been correlated with drug tolerance (9). It is not clear if the mutations that we see would affect the enzymatic activity of the protein or if the mutations may be in an enzyme binding site. However, it is of note that Rv1683 is one of three esterases active in the normoxia, hypoxia, and resuscitation phases of growth, underlining its importance (10). Future work should help to elucidate if one of these is the true target or if there are physiological changes that result in resistance.

In summary, we have identified a novel compound with efficacy against M. tuberculosis in both solid and liquid media that is also active against intracellular bacteria but with no cytotoxicity; thus, the profile of this compound is encouraging for future development. We have identified two routes to resistance to this compound in Rv1683 or Rv0047c and Rv3068c.

## Supplementary Material

Supplemental material
