# Supplementary material for: A Novel 6-Benzyl Ether Benzoxaborole Is Active against Mycobacterium tuberculosis In Vitro
Source: Antimicrob Agents Chemother. 2017 Aug 24;61(9):e01205-17. doi: 10.1128/AAC.01205-17 (PMC5571309; doi:10.1128/AAC.01205-17)
Supplement: Supplemental material [file supp_61_9_e01205-17__index.html]

Supplemental material 

# A Novel 6-Benzyl Ether Benzoxaborole Is Active against Mycobacterium tuberculosis *In Vitro*

## Supplemental material

- Supplemental file 1 -

  Text S1

  PDF, 91K
