# Supplementary material for: A Novel 6-Benzyl Ether Benzoxaborole Is Active against Mycobacterium tuberculosis In Vitro
Source: Antimicrob Agents Chemother. 2017 Aug 24;61(9):e01205-17. doi: 10.1128/AAC.01205-17 (PMC5571309; doi:10.1128/AAC.01205-17)
Supplement: Supplemental material [file AAC.01205-17_zac009176509s1.pdf]

Synthesis and characterization of 6-(benzyloxy)-4,7-dimethylbenzo[c][1,2]oxaborol-1(3H)-ol

To a solution of 5-methylbenzene-1,3-diol (8.0 g, 65 mmol) and DIPEA (48 mL, 325 mmol) in DCM (250 mL) was added chloromethyl ethyl ether (15 mL, 163 mmol) dropwise at room temperature and the mixture was stirred overnight. Water (100 mL) was added and the mixture was extracted with DCM (3 x 100 mL). The organic layers were washed with brine, dried over Na_2SO_4, filtered and concentrated to give 1,3-bis(ethoxymethoxy)-5-methylbenzene (13.0 g, yield 83%) as a colorless oil. MS: m/z = 241.3 (M+1, ESI+). To a solution of 1,3-bis(ethoxymethoxy)-5-methylbenzene (13.0 g, 54 mmol) in THF (200 mL) at 0°C under nitrogen was added dropwise n-BuLi (23.8 mL of a 2.5M solution in hexane, 60 mmol). The resulting suspension was warmed to 18°C and stirred slowly at this temperature for 1.5 h, and then treated with dry DMF (5 mL, 65 mmol). The resulting mixture was poured into water (100 mL) and extracted with diethyl ether (3 x 100mL). The combined organic phases were then washed with water (40 mL) and brine (40 mL), dried over Na_2SO_4, filtered, and concentrated under reduced pressure. The residue was purified by column chromatography on silica gel by elution with PE: EA=5:1 to give 2,6-bis(ethoxymethoxy)-4-methylbenzaldehyde (10.5g, yield 72%) as a pale-yellow solid. ^{1}H NMR (400 MHz, $CDCl_3$): δ 10.46 (s, 1H), 6.67 (s, 2H), 5.30 (s, 4H), 3.76 (q, *J*=6.8 Hz, 4H), 2.36 (s, 3H), 1.23 (t, *J*=6.8 Hz, 6H) ppm; MS: m/z = 269.0 (M+1, ESI+). To a solution of 2,6-bis(ethoxymethoxy)-4-methylbenzaldehyde (10.5 g, 39 mmol) in THF (120 mL) was added 4 N HCl in 1,4-dioxane (40 mL). Then the mixture was stirred overnight at room temperature. After removal of solvent, the residue was purified by Combiflash to give 2,6-dihydroxy-4-methylbenzaldehyde (2.9 g, yield 50%) as a yellow solid. ^{1}H NMR (400 MHz, CD_3OD): δ 10.23 (s, 1H), 6.17 (s, 2H), 2.24 (s, 3H) ppm; MS: m/z = 153.2 (M+1, ESI+). To a solution of 2,6-dihydroxy-4-methylbenzaldehyde (2.9 g, 19 mmol) and sodium cyanoborohydride (3.6 g, 57 mmol) in 80 mL of THF was added methyl orange as an indicator giving the solution a yellow color. Aqueous 1N HCl solution (57mL) was slowly added to the reaction system maintaining the color of orange. The mixture was stirred at room temperature for 3 h. Water was added, and the mixture was extracted three times with Et_2O. After removal of solvent, the residue was purified by Combiflash to give 2,5-dimethylbenzene-1,3-diol (1.0 g, yield 40%) as a white solid. ^{1}H NMR (400 MHz, DMSO-d_6): δ 8.87 (br. s, 2H), 6.08 (s, 2H), 2.07 (s, 3H), 1.87 (s, 3H) ppm; MS: m/z = 139.3 (M+1, ESI+). Phosphorous oxychloride (1.6

mL, 18 mmol) was added dropwise to DMF (7 mL) stirring at 0°C in a round-bottom flask under N_2 atmosphere for 30 min. The mixture was then transferred via cannula to a solution of 2,5-dimethylbenzene-1,3-diol (1.0 g, 7 mmol) in DMF (10 mL) stirring at 0°C in a round-bottom flask under N_2 atmosphere. The mixture was slowly warmed to room temperature and stirred overnight. The mixture was poured into ice water. Solid was precipitated out after 10 h and was collected by filtration to give 2,4-dihydroxy-3,6-dimethylbenzaldehyde (1.2 g, yield 60%) as a white solid. MS: m/z=167.2 (M+1, ESI+). A solution of 2,4-dihydroxy-3,6-dimethyl benzaldehyde (1.2 g, 7 mmol), $NaHCO_3$ (1.82 g, 21 mmol) and KI (240 mg, 1.4 mmol) in MeCN (40 mL) was slowly warmed to 60°C. BnBr (1.36 g, 8 mmol) was added at this temperature. The mixture was stirred overnight at 80°C. It was then cooled to room temperature, filtered and evaporated. The residue was mixed with water (20 mL) and extracted with EA (50 mL*2). The combined organic extracts were washed with brine, dried over Na_2SO_4, filtered and concentrated under a reduced pressure. The residue was purified by column chromatography on silica gel eluted with PE: EA=10:1 to give 4-(benzyloxy)-2-hydroxy-3,6-dimethylbenzaldehyde (1.48 g, yield 80%) as a white solid. MS: m/z = 257.3 (M+1, ESI+). To a solution of 4-(benzyloxy)-2-hydroxy-3,6-dimethylbenzaldehyde (1.48 g, 5.7 mmol) and Et_3N (2.92 g, 29 mmol) in DCM (40 mL) at 0°C was added a solution of Tf_2O (3.59 g, 12.7 mmol) in DCM (5 mL) dropwise. The reaction mixture was stirred at room temperature for 3 h. Water (50mL) was added and the mixture was extracted with DCM (50mL*2). The combined organic extracts were washed with brine, dried over Na_2SO_4, filtered and concentrated under a reduced pressure. The residue was purified by prep-HPLC to give 3-(benzyloxy)-6-formyl-2,5-dimethylphenyl trifluoromethane sulfonate (515 mg, yield 23%) as a white solid. MS: m/z = 389.1 (M+1, ESI+). To a solution of 3-(benzyloxy)-6-formyl-2,5-dimethylphenyl trifluoromethane sulfonate (515 mg, 1.33 mmol), 5,5,5',5'-tetramethyl-2,2'-bi(1,3,2-dioxaborinane) (896 mg, 3.98 mmol) and KOAc (650 mg, 6.63 mmol) in 1,4-dioxane (30mL) was added $PdCl_2(dppf)_2$ (108 mg, 0.13 mmol). The reaction mixture was stirred at 90°C under argon atmosphere overnight. The solvent was removed and the residue was purified by column chromatography on silica gel eluted with PE: EA=6:1 to give 4-(benzyloxy)-2-(5,5-dimethyl-1,3,2-dioxaborinan-2-yl)-3,6-dimethylbenzaldehyde (320 mg, crude) as a yellow solid. It was used in next step without further purification. To a solution of crude 4-(benzyloxy)-2-(5,5-dimethyl-1,3,2-dioxaborinan-2-yl)-3,6-dimethylbenzaldehyde (320 mg, 0.9 mmol) in THF (15 mL) was added $NaBH_4$ (68 mg, 1.8 mmol). The reaction mixture was

stirred at room temperature for 3 h, and then to it was slowly added 3N HCl to pH=2. The reaction mixture was stirred at room temperature overnight. The solvent was evaporated and the residue was purified by Combiflash to give 6-(benzyloxy)-4,7-dimethylbenzo[c][1,2]oxaborol-1(3H)-ol (190mg, yield 53% over 2 steps) as a white solid. ^{1}H NMR (400 MHz, DMSO-d_6): δ 8.89 (s, 1H), 7.47-7.32 (m, 5H), 6.97 (s, 1H), 5.10 (s, 2H), 4.84 (s, 2H), 2.30 (s, 3H), 2.16 (s, 3H) ppm; HPLC purity: 99.6% at 220 nm and 96.4% at 254 nm; MS: m/z = 267 (M-1, ESI$^-$).
